# A fusion of *CD63–BCAR4* identified in lung adenocarcinoma promotes tumorigenicity and metastasis

**DOI:** 10.1038/s41416-020-01146-3

**Published:** 2020-11-18

**Authors:** Kieun Bae, Jin Hee Kim, Hyojik Jung, Sun-Young Kong, Yun-Hee Kim, Sunshin Kim, Geon Kook Lee, Jin Soo Lee, Jake June-Koo Lee, Young Seok Ju, Yang-Kyu Choi, Kyong-Ah Yoon

**Affiliations:** 1 College of Veterinary Medicine, Konkuk University, Seoul, 05029 Republic of Korea; 2 College of Health Science, Cheongju University, Cheongju, 28503 Republic of Korea; 3 National Cancer Center Graduate School of Cancer Science and Policy, Goyang, 10408 Gyeonggi Republic of Korea; 4 Research Institute, National Cancer Center, Goyang, 10408 Gyeonggi Republic of Korea; 5 Hospital, National Cancer Center, Goyang, 10408 Gyeonggi Republic of Korea; 6 Graduate School of Medical Science and Engineering, Korea Advanced Institute of Science and Technology, Daejeon, 34141 Republic of Korea; 7 Department of Biomedical Informatics, Harvard Medical School, Boston, MA 02115 USA

**Keywords:** Non-small-cell lung cancer, Oncogenes

## Abstract

**Background:**

Recently, fusion variants of the breast cancer anti-oestrogen-resistance 4 *(BCAR4)* gene were recurrently discovered in lung adenocarcinoma from the genome-wide studies. However, the functional characterisation of BCAR4 fusion has not been investigated.

**Methods:**

Based on the analysis of RNA-sequencing data, we identified a fusion transcript of *CD63–BCAR4* in a Korean patient with lung adenocarcinoma who did not harbour any known activating mutations in *EGFR* and *KRAS* genes. To investigate the oncogenic effect of CD63–BCAR4, in vitro and in vivo animal experiments were performed.

**Results:**

In vitro experiments showed strongly enhanced cell migration and proliferation by the exogenous expression of CD63–BCAR4 protein in bronchial epithelial cells. Cell migration was notably reduced after knockdown of *BCAR4* fusion by small-interfering RNA. The tumorigenic and metastatic capability of the CD63–BCAR4 fusion was confirmed by using the mouse xenograft model. Fusion-overexpressed cells result in metastasis to the liver and lung as well as the primary tumours after subcutaneous injection into mice. Cyclin D1, MMP1, Slug and mesenchymal markers were significantly increased after *CD63–BCAR4* overexpression in the in vitro and in vivo experiments.

**Conclusions:**

Taken together, our results suggest a newly identified fusion gene, *CD63–BCAR4* as a potential novel oncogene in lung adenocarcinoma.

## Background

Patients with lung adenocarcinoma are classified based on molecular alterations of genes encoding kinases such as *EGFR*, *KRAS*, *ALK*, *ROS1* and *BRAF*.^1,2^ The presence of these alterations has led to clinical benefits for the subset of patients who can be effectively treated with targeted therapy. In addition, oncogenic fusion is an important genetic alteration that can drive lung cancer. A fusion gene comprising the echinoderm microtubule-associated protein-like 4 *(EML4)* and anaplastic lymphoma kinase *(ALK)* was discovered in non-small-cell lung cancer (NSCLC);^3,4^ subsequently, chromosomal rearrangements of *ALK, RET* and *ROS1* have been identified as significant driver oncogenes.^5–7^ The patients harbouring these therapeutically actionable fusion genes have experienced clinical benefits because of the development of effective targeted therapies, led by crizotinib.^8,9^ Recently, additional rearrangements of Neuregulin-1 (*NRG1*) were demonstrated in invasive mucinous adenocarcinomas of the lung, where they stimulate ERBB2/3 signalling.^10,11^ Recurrent fusions that were identified as drivers for lung cancer share a common factor: all are activating kinases with an intact kinase domain. The genomic landscape of lung adenocarcinoma has revealed that complex genomic rearrangements generated driver fusion oncogenes in smoking-signature-low patients.^12^

Based on the analysis of fusion transcripts, we tried to identify novel driver alterations in driver-negative lung cancers lacking therapeutically actionable alterations. RNA-sequencing analysis detected a novel fusion transcript of *CD63–BCAR4* in a patient with lung adenocarcinoma.

*BCAR4* was identified from functional screening for the genes responsible for tamoxifen resistance in breast cancer cells;^13^ notably, *BCAR4*-positive breast cancer demonstrated aggressive phenotypes and resistance to treatment.^14–16^ In its function as a lncRNA, *BCAR4* promotes the metastasis of breast cancer by activating cell migration via a non-canonical Hedgehog/GLI2 signalling pathway. LncRNA *BCAR4* interacts with Smad nuclear-interacting protein 1 (*SNIP1*) and serine/threonine-protein phosphatase 1 regulatory subunit 10 (*PPP1R10*, also known as *PNUTS*) to increase histone acetylation, thereby leading to the transcription of migration-related genes.^17^ Although *BCAR4* is a lncRNA, it has a short coding region on exon 4 that encodes a protein comprising 122 amino acids. Upregulated *BCAR4* has been reported as a marker for poor prognosis in colon cancer, gastric cancer and cervical cancer.^18–20^ Functional studies suggested *BCAR4* as a potential oncogene in breast, lung and brain cancers demonstrating enhanced proliferation and tumour formation by BCAR4.^14,21,22^

In this study, we investigated the oncogenic features of a fusion gene encoding a 211-amino-acid protein comprising exons 1–3 of *CD63* and exon 4 of *BCAR4*. The expression of *CD63–BCAR4* in human bronchial epithelial BEAS-2B cells induced dramatic enhancements in cell migration and consequent metastasis. Our study demonstrates the oncogenic activity of *CD63–BCAR4* that is newly identified in lung adenocarcinoma, with accompanying functional investigation.

## Methods

### Sample preparation and transcriptome analysis

This study used surgically resected tissue specimens of 29 patients with lung adenocarcinoma that were stored in the tumour tissue bank of National Cancer Center Korea as approved by the Institutional Review Board (IRB No. NCC-2014-0115). Genomic DNA and RNA were isolated from tumour and adjacent normal tissues by using the AllPrep DNA/RNA mini kit (Qiagen, Valencia, CA, USA) and TRIzol reagent (Life Technologies, Carlsbad, CA, USA), in accordance with the manufacturers’ protocols. RNA sequencing was performed with cDNA libraries prepared from RNA by using the Illumina TruSeq sample preparation kit. The sequencing was performed on an Illumina HiSeq 2500 platform (Illumina, San Diego, CA, USA), thereby generating paired-end 2 × 100-bp reads. Fusion calling was performed using ChimeraScan, DeFuse and FusionMap.^23–25^ The fusions were detected by analysing the discordant and split reads mapped to different genes. Fusion candidates with multiple partner genes, or without in-frame coding regions, were eliminated based on the criteria as described in the fusion Database (www.tumorfusions.org).^26^ Fusion transcripts that showed low fusion-spanning reads (<5) and low-split reads (<5) were also excluded. Fusion candidates that were identified by at least two caller programs were selected for further analysis. The fusion junction was confirmed by RT-PCR and Sanger sequencing using RNA from the tumour tissue of the index case.

### Cell culture and stable cell lines

The presence of *CD63–BCAR4* fusion transcript and expression of *BCAR4* were examined in 24 cell lines. Lung cancer cell lines were purchased from Korean cell line bank (KCLB, Seoul, Korea) (NCI-H187, NCI-H417, NCI-H1299, NCI-H596, NCI-H146, SNU-1327, Hcc1195, Hcc1171, NCI-H1703, NCI-H69, Hcc95, Hcc2108, Hcc2279, A427, A549, NCI-H23, NCI-H460 and NCI-H358) and American type culture collection (ATCC, Manassas, VA, USA) (BEAS-2B, NCI-H226, NCI-H2171, NCI-H1395, NCI-H1975 and NCI-H2228). Immortalised human bronchial epithelial cell line BEAS-2B and breast cancer cell line MDA-MB-231 were purchased from ATCC. Each cell line was cultured in the appropriate medium with 10% foetal bovine serum and 1% penicillin–streptomycin or airway epithelial cell basal medium with bronchial epithelial cell growth kit. The authentication of the cell lines was assessed by using short tandem repeat analysis and mycoplasma infection was tested by PCR. Coding regions of the *CD63–BCAR4* fusion and *BCAR4* (HE601934.1) were cloned into a lentiviral vector with hemagglutinin (HA) tag, CMV promoter and green fluorescent protein (GFP) as a transduction marker. To establish stable cell lines for *CD63–BCAR4*, *BCAR4* and empty vector, the infected cells were sorted by GFP signal intensity.

### Transfection of siRNAs

The small-interfering RNAs (siRNAs) that were pre-designed and verified to target BCAR4 (Hs.24611) and Silencer^®^ negative control No.1 (AM4635) were purchased from Invitrogen (Carlsbad, CA, USA). The siRNA-targeting BCAR4 (si-BCAR4) and non-targeting negative control (si-control) were transfected into cells with lipofectamine 2000 reagent (Invitrogen) following the manufacturer’s instructions. After 48 h of transfection with the siRNAs (0.1 nmol/2 × 10^5^ cells), the cells were harvested for further experiments.

### Cell growth and colony-formation assay

Growth rates of stable cell lines were compared using the IncuCyte™ Live-Cell Imaging System (IncuCyte live-cell ESSEN BioScience Inc., Ann Arbor, MI, USA) in a 96-well Essen ImageLock plate. Colony-forming ability was measured for 2 weeks after 200 cells/well were seeded in 6-well plates. The number of colonies was counted after staining with 0.5% crystal violet solution. All experiments were performed in triplicate.

Cells were synchronised by the double-thymidine-block method as previously described.^27^ Briefly, cells (1.5 × 10^5^) in a 6-well plate were incubated in medium containing 2 mM thymidine (Sigma-Aldrich, St. Louis, MO, USA) for 16 h, released into normal medium for 9 h and then incubated for 16 h in medium containing 2 mM thymidine. All data were analysed with BD FACSCalibur (Becton Dickinson, San Jose, CA, USA) and using NovoExpress 1.2.1 software (ACEA Biosciences, Inc., San Diego, CA, USA).

### Migration assay

After wounds were made on the confluent monolayer of cells using the 96-pin Wound Maker, cell migration was monitored with the IncuCyte™ Live-Cell Imaging System. Wound images were acquired by automatic scanning of the plates every 2 h, and the collected data were analysed by the IncuCyte software system. Transwell plates (Corning, Inc., Corning, NY, USA) were also used to test migration ability. Cells were seeded under serum-starved conditions in the upper chamber, while the bottom chamber was filled with medium supplemented with 1–5% FBS. After incubation at 37 °C with 5% CO_2_ for 24 h, the migrated cells were stained with crystal violet. Each experiment was performed at least three times.

### Western blot analysis and immunohistochemistry (IHC)

Total protein was extracted from cell lines or tissues by using RIPA Lysis Buffer (Cell Signaling, Danvers, MA, USA), in accordance with the manufacturer’s instructions. After quantification using Protein assay solution (Bio-Rad, Hercules, CA, USA), immunoblotting was performed according to the standard procedure. Primary antibodies for E-cadherin, N-cadherin, Slug, Cyclin D1 and HA tag were purchased from Cell Signaling Technology. Proteins were detected with horseradish peroxidase-conjugated anti-mouse or anti-rabbit antibodies. Enhanced chemiluminescence reagent (GE Healthcare Life Sciences, Marlborough, MA, USA) was used to visualise the protein bands. β-actin was used as the loading control.

The resected tissues from the xenografted mice were fixed in 4% paraformaldehyde and embedded in paraffin. Tissue sections were stained with haematoxylin and eosin (H&E) solutions or with specific antibodies for immunohistochemistry. After incubation with biotinylated secondary antibodies, the tissues were processed with 3,3′-diaminobenzidine substrate for enzymatic detection.

### Microarray and RT-PCR

Total RNA of each cell line was extracted using TRIzol reagent (Invitrogen) and was subjected to cDNA microarray. Microarray experiments were performed using the commercial service that was provided by Ebiogen, Inc. (Seoul, Korea). Isolated RNA was used to synthesise cDNA and amplified cRNA for hybridisation to the Agilent Human Gene Expression 4X 44K Microarray (Agilent Technologies, Palo Alto, CA, USA) according to the manufacturer’s instruction. Gene expression was quantified from the hybridisation images using Agilent Feature Extraction software 10.7 (Agilent Technologies, USA). Data normalisation and the selection of fold-changed genes were performed using GeneSpringGX 7.3.1 (Agilent Technologies). The functional annotation of the genes was performed according to the Gene Ontology Consortium (http://www.geneontology.org) and the QuickGO Database (https://www.ebi.ac.uk/QuickGO/).^28^

For RT-PCR experiment, total RNA was reverse-transcribed into cDNA using the two-step RT-PCR kit (Invitrogen). Quantitative real-time polymerase chain reaction (qRT-PCR) was performed by using the SYBR Select Master Mix (Bio-Rad). The relative quantitative value was expressed by the 2^−ΔCt^ method using glyceraldehyde-3-phosphate dehydrogenase (*GAPDH*) as control. Each experiment was performed in triplicate and repeated more than twice.

### In vivo tumorigenicity

In vivo tumorigenicity was tested by injecting BEAS-2B cells (5 × 10^6^) subcutaneously into 6-week-old NOD.Cg-Prkdcscid II2rgtm1Sug/Jic (NOG) mice (average body weight 18 g ± 1.5 g) after induction of anaesthesia by 1.5–3% isoflurane inhalation (HanaPharm, Seoul, Korea). Mice were purchased from Central Institute for Experimental Animals (CIEA, Tokyo, Japan) and housed 4–5 per cage in the animal facility under the controlled temperature (22–24 °C) and humidity (40–60%). Tumours and body weight were measured weekly, and tumour volumes were calculated (length × width^2^ × 0.5)^29^ to compare tumorigenicity between BCAR4 fusion and control (eight mice per group). After 5 months, the mice were sacrificed with carbon dioxide gas before tumours reached 1,500 mm^3^ in volume. Tumours and other organs were harvested for further molecular experiments. To dissociate cells, the tumours were minced into 2–4-mm fragments, which were then incubated with the appropriate dissociation solutions containing collagenase II (1.5 mg/ml) (Life Technologies), hyaluronidase (20 µg/ml) and Ly27632 (10 µM) enzymes (Sigma-Aldrich, St. Louis, MO, USA), for 30 min at 37 °C as described by Petit V. et al.^30^ After treatment with enzymes, the dissociated cells were filtered through a 40-μm nylon mesh cell strainer (BD Biosciences, San Diego, CA, USA). The filtered cells were centrifuged and cultured in media with 10% FBS at 37 °C. All animal experiments were performed under the approval of the Institutional Animal Care and Use Committee (IACUC) of our institute (NCC-15-271, NCC-19-496 and KU17167).

### Statistical analysis

Statistical significance was tested by unpaired *t* test or one-way ANOVA for comparison between two or more groups. All reported *P* values are two-sided, and the graphs were generated with GraphPad Prism software (GraphPad Software Inc., San Diego, CA, USA). Significant differences are marked with *(*p* < 0.05), **(*p* < 0.005) and ***(*p* < 0.0005) in the figures.

## Results

### A fusion transcript of *CD63–BCAR4* is discovered in a patient with lung adenocarcinoma

**Fig. 1:**
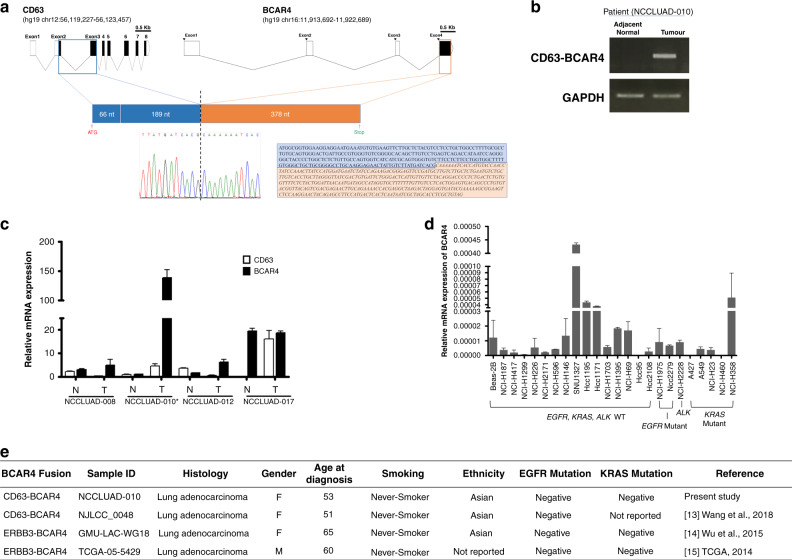
Discovery of the *CD63–BCAR4* fusion gene in a patient with lung adenocarcinoma. **a** Schematic representations of the wild-type *CD63* and *BCAR4* genes with breakpoints of the respective exon numbers and *CD63–BCAR4* fusion. The sequencing chromatogram shows the breakpoint, confirmed by Sanger sequencing. **b** Expression of fusion gene of *CD63–BCAR4* was confirmed by RT-PCR in the tumour tissue of a positive patient (NCCLUAD-010). **c** High expression of *BCAR4* mRNA in the index case. Quantitative real-time RT-PCR was performed to compare mRNA expression of *BCAR4* and *CD63* in tumour (T) and adjacent normal (N) tissues from four patients. * Indicates the index case. **d** Expression of BCAR4 mRNA in lung cancer cell lines showing mutation status of EGFR, KRAS and ALK genes. **e** Fusion transcripts of BCAR4 that were detected in the patients with lung adenocarcinoma. References were indicated for previously reported fusions.

To investigate fusion genes in lung cancer, we analysed RNA-sequencing data of 29 patients with lung adenocarcinoma (NCCLUAD) who lacked activating mutations in *KRAS* or *EGFR* (Supplementary Table 1). No patients harboured somatic mutations on exons 18–22 of *EGFR* or exon 1 of *KRAS*, as confirmed by Sanger sequencing and digital PCR analysis. RNA-sequencing analysis revealed that 5 patients harboured *EML4–ALK* fusions, three patients harboured *KIF5B–RET* fusions and one patient harboured a *ROS1* fusion. Among the remaining patients, we detected a patient with a novel fusion transcript of *CD63–BCAR4*. This fusion was an inter-chromosomal fusion comprising exons 1–3 of *CD63* on chromosome 12 and the final exon of *BCAR4* on chromosome 16 (Supplementary Fig. 1). The junction sequences of the fusion transcript were confirmed by Sanger sequencing analysis (Fig. 1a). A coding region was predicted, showing translation initiation from an ATG site in the *CD63* gene (Hs.445570) that fused to *BCAR4*, producing a 211-amino-acid protein. The CD63–BCAR4 fusion comprises 85 amino acids of CD63 and 126 amino acids from BCAR4 in-frame. The expression of this fusion gene was highly upregulated only in tumour tissues of the patient, but was not present in adjacent normal tissues (Fig. 1b). The index case carrying CD63–BCAR4 fusion also showed highly upregulated *BCAR4* mRNA expression in tumour tissue when compared with other patients (Fig. 1c). BCAR4 fusion was not detected in lung cancer cell lines; however, high expression of *BCAR4* was detected in lung cancer cell lines SNU-1327, HCC-1195, HCC-1171 and NCI-H358 as shown in Fig. 1d. This fusion was noticeable because the recurrent fusions of *BCAR4* were reported by recent genomic analyses of lung adenocarcinoma. Recently, a fusion transcript of *CD63–BCAR4* was also reported in a comprehensive genomic study of Chinese NSCLC patients.^31^ Fusion variants of *ERBB3–BCAR4* were detected in two patients, one from a whole-genome-sequencing dataset of Chinese lung adenocarcinoma patients and another one from lung adenocarcinoma project of The Cancer Genome Atlas consortium (TCGA–LUAD) as demonstrated in Fig. 1e.^32,33^

Fusion gene of CD63–BCAR4 was cloned from the index case. *BCAR4* is known as breast cancer anti-oestrogen-resistance 4, and was recently reported as a metastasis-stimulating lncRNA in breast cancer.^17,34^ However, the oncogenic effects of *BCAR4* fusion in lung cancer have not been identified.

### CD63–BCAR4 fusion increases cell proliferation and colony formation

**Fig. 2:**
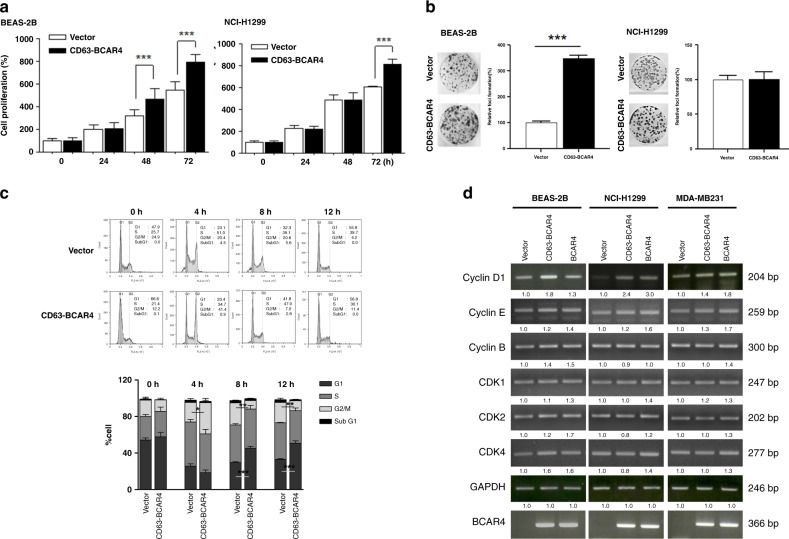
Increased cell proliferation and colony formation by CD63–BCAR4. **a** Relative cell growth (%) of BEAS-2B cells expressing empty vector (Vector) or CD63–BCAR4. Cell proliferation of BEAS-2B (bronchial epithelial cell line) and NCI-H1299 (lung cancer cell line) was measured using the IncuCyte™ Live-Cell Imaging System. **b** Representative colonies and relative colony-forming activity of *CD63–BCAR4* or empty vector-expressing cells. **c** Representative DNA histograms and bar graph of cell-cycle distribution at the indicated time points after a double-thymidine block. Overexpressed BEAS-2B cells were synchronised in G1 by a double-thymidine block, and then released into the cell cycle for the times indicated. Each bar in the graph indicates the average cell-cycle distribution from triplicated experiments. **d** Expression levels of cell-cycle-related genes after exogenous expression of *CD63–BCAR4* and *BCAR4*. The relative expression was compared to the control gene GAPDH by using semi-quantitative RT-PCR. Data in this figure are presented as the representative results of three independent experiments. Bar graphs demonstrated the mean ± SD. Statistically significant differences are marked with *(*p* < 0.05), ** (*p* < 0.005) and ***(*p* < 0.0005), respectively.

Overexpression of CD63–BCAR4 increased cell proliferation compared to the empty vector in immortalised normal bronchial epithelial cells (BEAS-2B) and lung cancer cells (H1299) (Fig. 2a). Additionally, the exogenous expression of *CD63–BCAR4* enhanced colony formation compared to the control vector in BEAS-2B cells (Fig. 2b). The increased colony-forming activity by BCAR4 fusion was also shown in A549 and MDA-MB-231 cells (Supplementary Fig. 2). To analyse the effects of fusion on the cell cycle, stably overexpressed cells were synchronised at the G1/S phase using a double-thymidine block, and then released into the cell cycle as determined by flow cytometric analysis. A larger portion of fusion-overexpressed cells showed the exit from G1 and the entry into G2/M at 4 h after the thymidine block compared to control cells (20.4% vs. 41.4%). In addition, the cell cycles recovered from thymidine-double block more rapidly in fusion-overexpressed than control cells (Fig. 2c). The exogenous expression of CD63–BCAR4 resulted in increased mRNA level of Cyclin D1 in BEAS-2B and H1299 cells (Fig. 2d). Related cyclins and CDKs were not changed by overexpression of BCAR4 and CD63–BCAR4.

### Cell migration is promoted by *CD63–BCAR4* fusion

**Fig. 3:**
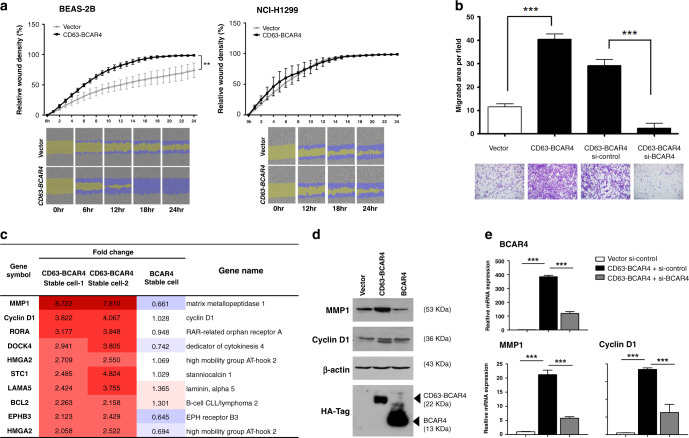
Effects of *CD63–BCAR4* expression on cell migration and gene expression. **a** Promoted cell migration by CD63–BCAR4 in BEAS-2B cells. Wound-healing assay was used to compare the migration of CD63–BCAR4-overexpressing BEAS-2B and H1299 cells compared to vector- overexpressing cells. **b** Migration of *CD63–BCAR4*-overexpressing cells and BEAS-2B cells in which the fusion gene was suppressed by siRNA. **c** Upregulated genes by overexpression of CD63–BCAR4 in BEAS-2B cells. Fold changes of differentially expressed genes were analysed by RNA microarray. **d** Enhanced protein levels by CD63–BCAR4 fusion were examined in BEAS-2B cells by western blot analysis. **e** Gene expression levels after transfection of siRNA targeting BCAR4 and negative control. Expression was assessed in BEAS-2B cells by real-time RT-PCR. In each experiment, the results are demonstrated with representative data from the replicated experiments and statistical significances are marked with *(*p* < 0.05), **(*p* < 0.005) and ***(*p* < 0.0005).

The overexpression of *CD63–BCAR4* in BEAS-2B cells significantly enhanced migration as shown by the wound-healing and the transwell assay (Fig. 3a, b). BEAS-2B cells, which are normal epithelial cells, showed augmented migration by CD63–BCAR4 expression compared to lung cancer cells, H1299. The enhanced migration was reversed by transiently transfected siRNA targeting *CD63–BCAR4* (Fig. 3b). Differentially expressed genes were screened by using microarray and selected based on fold changes (≥2 or ≤0.5) in two stable cell lines expressing CD63–BCAR4 (Supplementary Table 2). Genes related to cell proliferation and migration were filtered, and the expression of selected genes was verified by RT-PCR using specific primers (Fig. 3c and Supplementary Fig. 3). Microarray data and qRT-PCR revealed significant increase in *MMP1* and *Cyclin D1* mRNA expression in fusion-overexpressed cells (Fig. 3c, e). Upregulated MMP1 and Cyclin D1 proteins were also demonstrated in CD63–BCAR4-overexpressing cells by western blot analysis (Fig. 3d). Knockdown of the fusion gene by transfected siRNA targeting BCAR4 reversed the expression of *MMP1* and *Cyclin D1* (Fig. 3e). These results indicate that exogenous *CD63–BCAR4* increased MMP1 and Cyclin D1 expression.

### Exogenous CD63–BCAR4 fusion protein induces tumorigenicity and metastasis

**Fig. 4:**
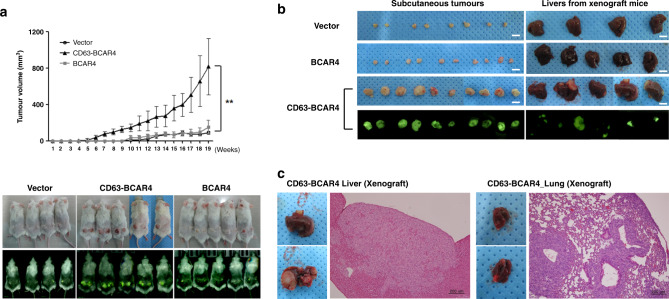
Tumorigenicity of CD63–BCAR4 fusion. **a** Tumour formation over 19 weeks in NOG mice transplanted with vector, CD63–BCAR4 or BCAR4-overexpressing BEAS-2B cells. Tumour growth was monitored after the subcutaneous injection of stable cell lines to NOG mice. The results are shown as the means ± SD (*n* = 8/group). Statistical significances are marked with **(*p* < 0.005). The representative image of the xenografted mice is demonstrated. GFP protein was used as a transduction marker of lentiviral vector. **b** Resected tumour specimens and liver from the subcutaneously transplanted mice. **c** H&E-stained tissues from metastatic tumours in the liver (front, back) and lung (front, back) of the xenograft mice injected with CD63–BCAR4-overexpressing cells.

**Fig. 5:**
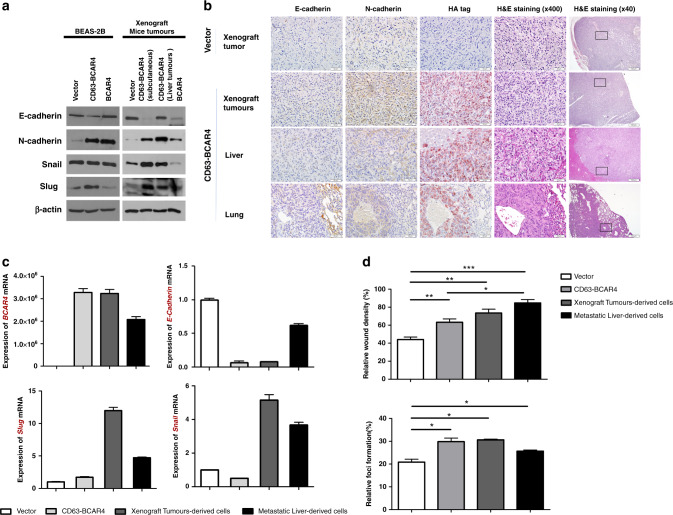
Effects of *CD63–BCAR4* on the expression of EMT-related genes. **a** Protein expression in overexpressed BEAS-2B cells and tumour tissues from the xenograft mice. **b** IHC of E-cadherin, N-cadherin, HA protein and H&E staining in tumour sections from the xenografted mice. **c** Expression of EMT-related genes in stable cell lines and xenografted mice-derived cells. mRNA expression of genes was examined in overexpressed and xenograft-derived cells by real-time RT-PCR. **d** Migration and colony-forming activity of stable cell lines and tumour-dissociated cells from xenograft tumour and metastatic liver. Statistical significances are marked with *(*p* < 0.05), **(*p* < 0.005) and ***(*p* < 0.0005). All experiments were repeated three times independently.

In vivo tumorigenicity of the CD63–BCAR4 fusion protein was examined by using the mouse xenograft model with stable cell lines. Tumour formation was observed 6 weeks after the subcutaneous injection of CD63–BCAR4-overexpressing BEAS-2B cells to NOG mice. Tumour growth was faster in the mice after the subcutaneous injection of CD63–BCAR4-overexpressing cells compared to the empty vector-overexpressed cells (Fig. 4a). Moreover, we found tumour masses in the liver and lung of the NOG mice subcutaneously injected with CD63–BCAR4-overexpressing BEAS-2B cells (Fig. 4b, c). The invasive feature of tumour cells was confirmed by H&E staining of tumours resected from the liver and lungs of the xenografted mice. CD63–BCAR4 fusion induced tumour mass in the liver of all the xenografted mice (*n* = 8), whereas the control cells expressing empty vector did not form any tumours in the liver or lung of NOG mice (*n* = 8). Expression of CD63–BCAR4 in tumours was supported by the bioimaging of GFP protein, a transduction marker of the lentiviral vector. Immunohistochemical analysis for HA-tagged fusion protein confirmed overexpression of CD63–BCAR4 in tumour cells of subcutaneous tumours, as well as metastatic tumours in the liver and lung of xenografted mice (Fig. 5b). We also found tumours in the liver and lungs in NOG mice 12 weeks after intravenous injection of CD63–BCAR4-overexpressing cells into the tail vein (Supplementary Fig. 4).

### *CD63–BCAR4* fusion upregulates mesenchymal marker proteins

Since our in vitro and in vivo experiments indicated that promoting cellular migration as well as distant metastasis could be the phenotypic hallmark of *CD63–BCAR4* fusion, we further investigated the impact of this fusion gene on epithelial–mesenchymal transition (EMT). We identified a significant downregulation of E-cadherin and an upregulation of Slug and N-cadherin upon the overexpression of *CD63–BCAR4* fusion. Western blot analysis revealed that N-cadherin and Slug were highly upregulated by overexpression of *CD63–BCAR4* (Fig. 5a) in subcutaneous tumours from the xenografted mice as well as stable cell lines. The exogenous expression of fusion protein and upregulated N-cadherin in the xenografted tumours was also confirmed by IHC (Fig. 5b). Enhanced mRNA expression of *Slug* and *Snail* was also observed in subcutaneous tumours as well as fusion- overexpressing BEAS-2B cells (Fig. 5c). Subsequently, the migration ability and colony-forming activity were examined in BEAS-2B stable and xenograft-derived cells that were dissociated from subcutaneous and metastatic tumours. The dissociated tumour cells sustained the same pattern of promoted migration and colony formation as the fusion-overexpressed cells (Fig. 5d). These in vitro and in vivo experiments demonstrated that Cyclin D1, MMP1, Slug and N-cadherin were significantly increased after *CD63–BCAR4* overexpression.

## Discussion

We identified the tumorigenicity and metastatic capability of *CD63–BCAR4* as a novel fusion gene of lung cancer. A Korean female patient with lung adenocarcinoma who had no smoking history harboured this *CD63–BCAR4* fusion. Recently, a fusion transcript of *CD63–BCAR4* was also reported in a comprehensive genomic study of Chinese NSCLC patients.^31^ Wang et al. identified that a never-smoking female patient with lung adenocarcinoma harboured a *CD63–BCAR4* fusion, similar to the fusion described in the present study. The *CD63–BCAR4* fusion was expressed only in tumour tissue and not in the adjacent normal tissue. However, the tumorigenic feature of *CD63–BCAR4* has not yet been investigated. The reason to focus our attention on BCAR4 fusion was based on the discovery of a different fusion variant of *BCAR4* that was recently reported in additional patients with lung adenocarcinoma. One patient was identified from a whole-genome-sequencing dataset of Chinese lung adenocarcinoma patients and another one from lung adenocarcinoma project of The Cancer Genome Atlas consortium (TCGA–LUAD).^32,33^ These two patients harboured *ERBB3–BCAR4* fusion and had no other genetic alterations in canonical oncogenes. Interestingly, the BCAR4 fusion-positive patients showed the consistent features of being never-smokers, exhibiting absent or rare mutations of *EGFR* or *KRAS* genes and diagnosis with lung adenocarcinoma. In particular, out of 660 TCGA lung adenocarcinomas that were analysed in a previous study,^35^ 210 had low or moderate exposure to tobacco smoking (exposure to smoking-related mutational signature <0.2). Among these tumours, 44 had no apparent driver oncogenic alterations, and one was the tumour (TCGA-05-5429) harbouring *ERBB3*–*BCAR4* fusion. Therefore, among the never-smoker lung adenocarcinoma cases without any known driver alterations in oncogenes such as *EGFR*, *KRAS*, *ALK*, etc., the estimated fraction of *BCAR4* fusion could be approximately 2.2% (1/44). The frequency of *CD63–BCAR4* in lung adenocarcinoma without *EGFR* and *KRAS* mutations could be estimated as approximately 3% based on the present study (1/29) and Wang’s study (1/33); however, it might be much lower in Caucasians and/or in smokers. Apparently, these findings should be validated in further studies.

The function of *BCAR4* as a lncRNA was stimulated via a non-canonical Hedgehog/GLI signalling pathway. Although we examined the related genes, including *SNIP1, PPP1R10* and *PTCH1*, a *GLI* target gene, we could not find any change that was induced by BCAR4 fusion (Supplementary Fig. 5).

As the detected fusions of BCAR4 in lung cancer commonly included exon 4 of BCAR4, the effect of a short protein of BCAR4 was compared to CD63–BCAR4 fusion protein. We discovered an enhanced function of the CD63–BCAR4 fusion, compared with BCAR4 protein. The short BCAR4 protein could not induce tumorigenicity to a similar extent as its fusion form as demonstrated in xenografts of immortalised normal cells. Furthermore, only CD63–BCAR4 overexpression demonstrated dramatically enhanced cell migration and an increased number of metastatic nodes in the liver and lungs, as well as primary tumours in the xenograft models. These results confirmed that the fusion form of BCAR4 acquired the promoted tumorigenicity and metastatic activity than the BCAR4 protein. CD63 is the tetraspanin superfamily member;^36^ however, the tetraspanin domain of CD63 is not included in CD63–BCAR4 fusion. The potential functions of CD63 as a partner gene need to be investigated in further study.

We provide the results that the EMT signal was stimulated by CD63–BCAR4, not by BCAR4 protein, thus leading to enhanced metastasis. Increased Slug, Snail and N-cadherin were amplified in xenograft-derived tumours compared to stable cell lines. The EMT signal could be one of the potential mechanisms of BCAR4 fusion, and further studies need to be followed to elucidate the interacting proteins.

We could observe the recurrent fusions of BCAR4 in a total of four patients with lung adenocarcinoma and demonstrated the oncogenic effect of this novel fusion. The recurrent fusions of BCAR4 that were identified in lung adenocarcinoma without known drivers made it feasible to infer the oncogenic functions of BCAR4 fusion.

Therefore, our findings suggest that *BCAR4* rearrangements need to be surveyed in lung adenocarcinoma without identifiable driver oncogenes and may serve as a potential therapeutic avenue for the patients. Research is ongoing to screen therapeutic agents for cancer with *BCAR4* rearrangements.

In conclusion, our study provides evidence for tumorigenicity and metastasis induced by *CD63–BCAR4* fusion. We also suggest that lung adenocarcinomas harbouring *BCAR4* fusions might be a clinically relevant subgroup with a targetable oncogenic driver, among patients without *EGFR* and *KRAS* mutations.

## Supplementary information

Supplementary figures

Supplementary tables

## Data Availability

The datasets analysed in the present study are available from the corresponding author on reasonable request.
