# Supplementary material for: A fusion of CD63–BCAR4 identified in lung adenocarcinoma promotes tumorigenicity and metastasis
Source: Br J Cancer. 2020 Nov 18;124(1):290–8. doi: 10.1038/s41416-020-01146-3 (PMC7782829; doi:10.1038/s41416-020-01146-3)
Supplement: Supplementary file 1 — Supplementary figures [file 41416_2020_1146_MOESM1_ESM.pdf]

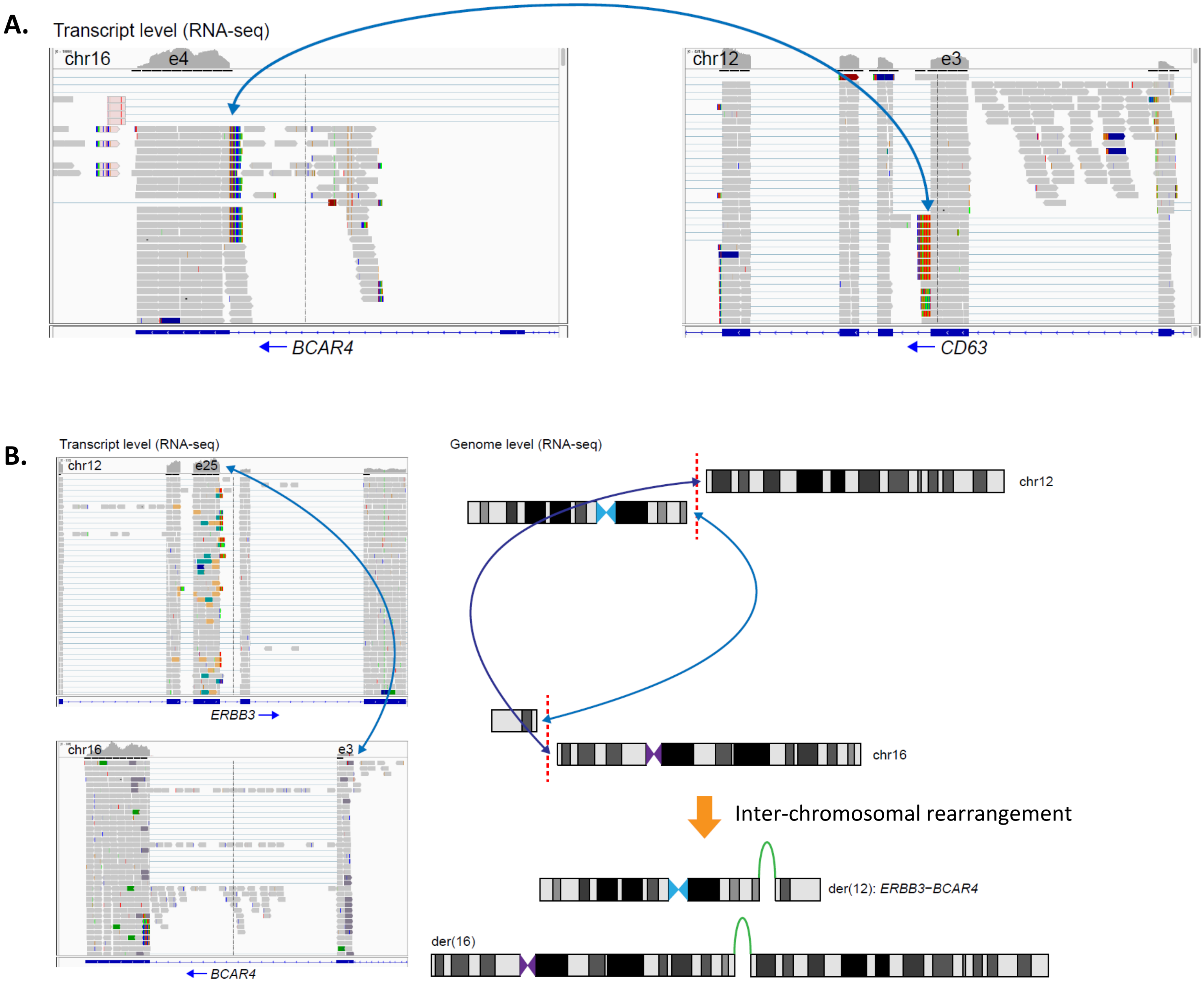

Supplementary Fig.1 BCAR4 fusions that discovered in lung adenocarcinomas. A. *CD63-BCAR4* fusion detected in a patient (NCCLUAD-010) in the present study. **B.** *ERBB3-BCAR4* fusion occurred in one TCGA patient

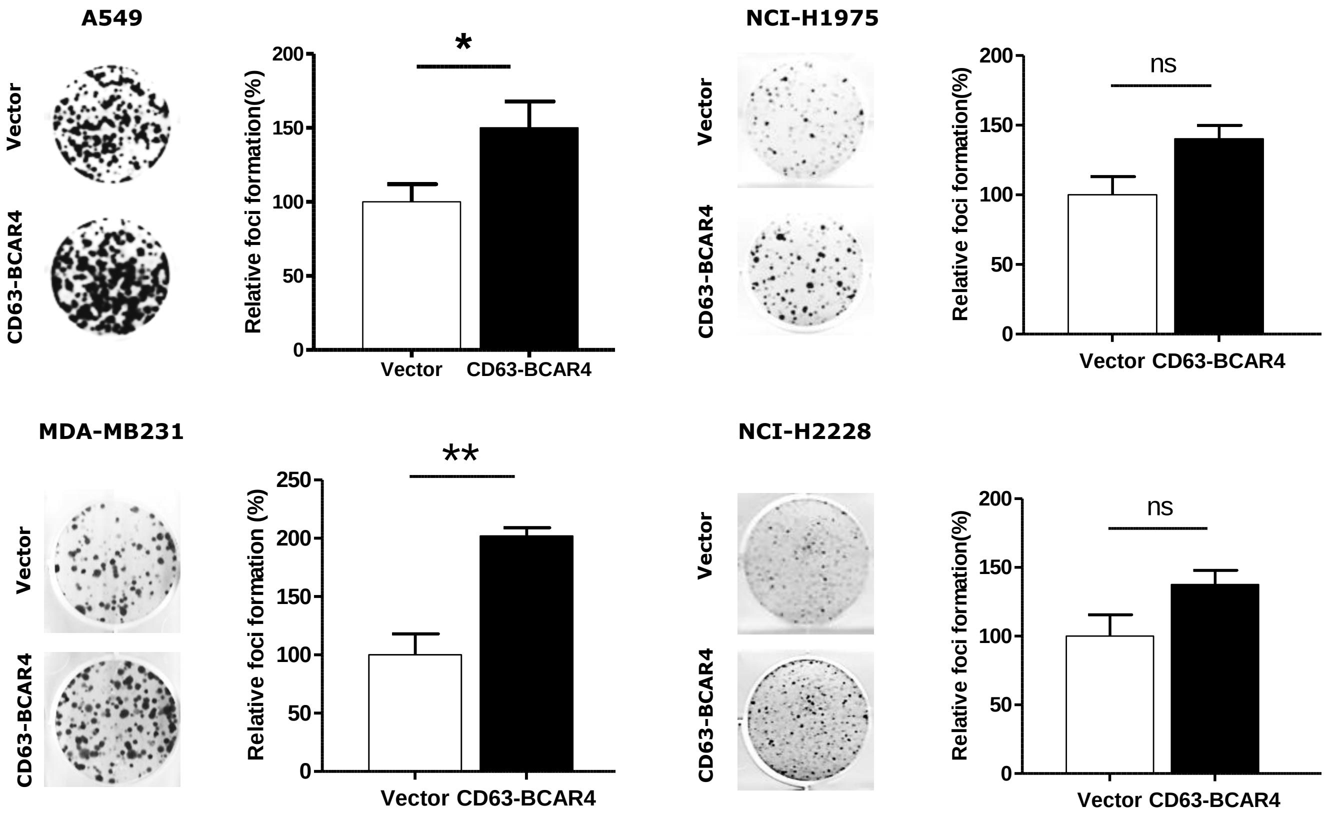

Supplementary Fig.2 Colony forming assay in lung and breast cancer cells overexpressing CD63-BCAR4. Colony-forming ability was measured for two weeks after 100 cells/well were seeded into 6-well plates. The number of colonies was counted after staining with 0.5% crystal violet solution. All experiments were performed in triplicate. Representative colonies and relative colony-forming activity of CD63-BCAR4 or empty vector-expressing cells. The statistical significances are marked with * ($p < 0.05$), ** ($p < 0.005$), or ns (not significant).

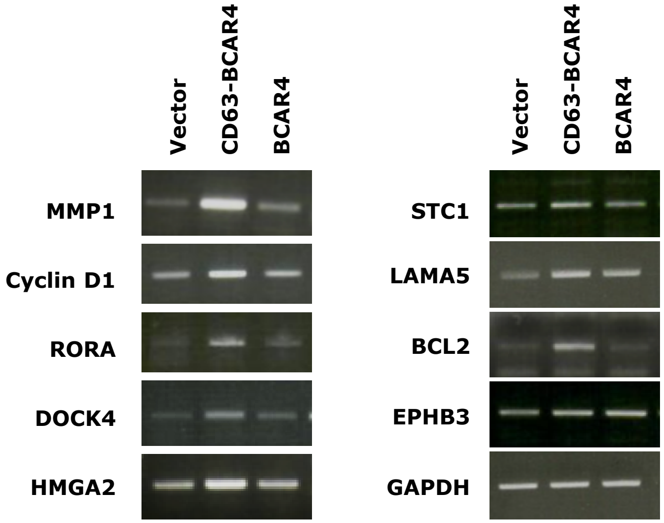

Supplementary Fig.3 ***Gene expression in BEAS-2B cells overexpressing CD63-BCAR4 and BCAR4.*** Semi-quantitative RT-PCR was performed to compare mRNA expression of genes that demonstrated differential expression in microarray.

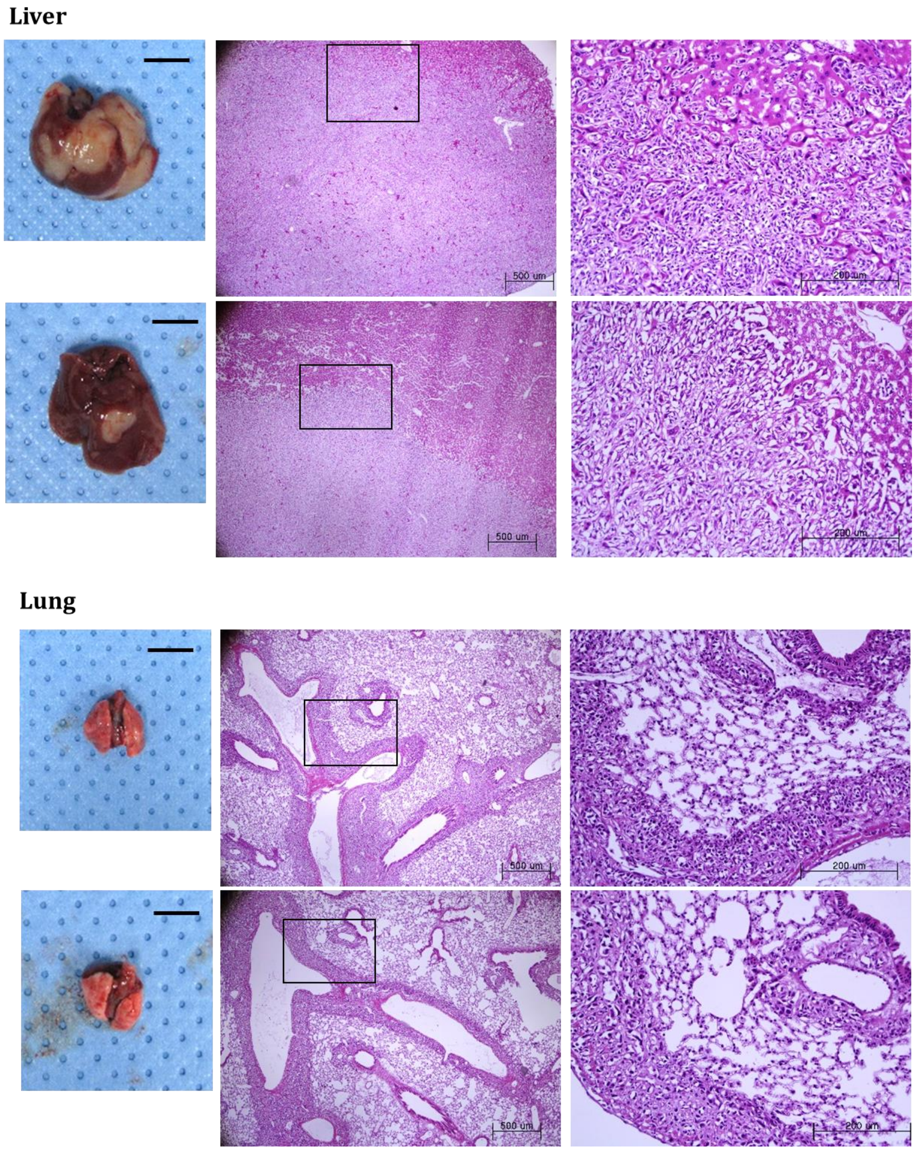

Supplementary Fig.4 Tumor formation in liver and lung after intravenous injection in NOG mice. CD63-BCAR4 overexpressing BEAS-2B cells (2×10^5) were injected into tail vein of NOG mice (n=3). Mice were housed under pathogen-free condition in the animal facility and monitored two times a week. Mice were sacrificed at three months after intravenous injection of cells to evaluate tumor growth in organs. Tissue sections of liver and lung were stained with H&E. All animal experiments were performed under the approval of the ethical committee (IACUC) of our institute (NCC-15-271 and KU17167).

A

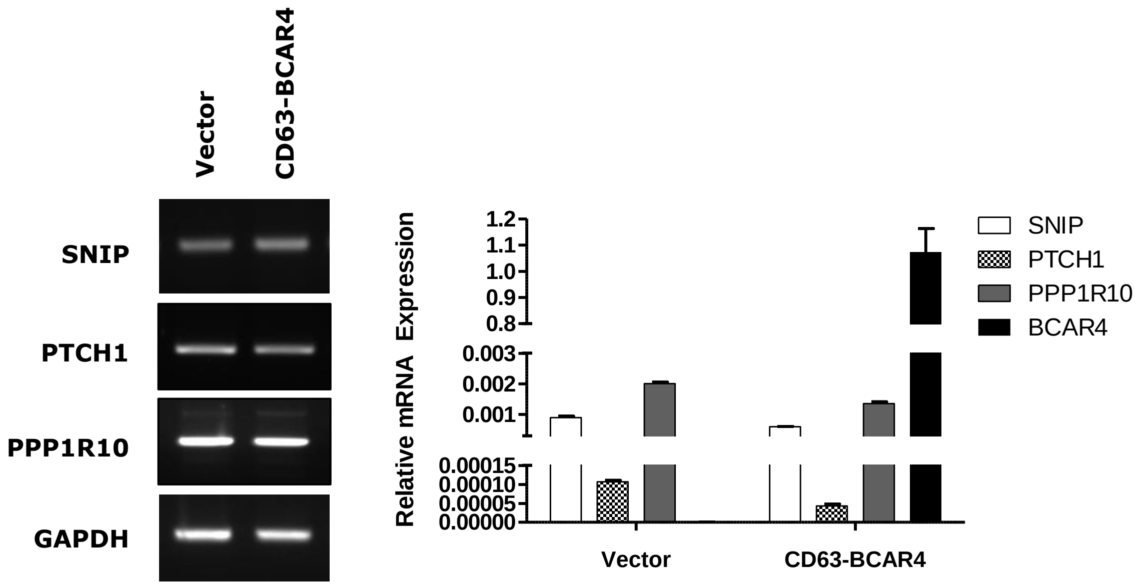

B

Gene Symbol	CD63-BCAR4 Stable cell-1	CD63-BCAR4 Stable cell-2	BCAR4 Stable cell
**BCAR4**	556.467	674.303	707.910
**BCL2**	2.263	2.158	1.301
	1.530	1.412	1.110
**GLI1**	0.962	1.538	1.099
**PTCH1**	1.119	1.010	0.769
	0.722	1.028	1.428
	0.798	1.760	1.694
**CTNNB1**	1.082	1.258	1.192
	1.069	1.218	1.160
**MMP13**	1.005	0.981	1.016
**FGF8**	1.005	0.981	1.016
**FGFR3**	0.594	0.781	0.624
	0.682	0.881	1.061
**STK3**	0.838	0.935	0.870
	0.902	1.041	1.084
**SNAI1**	0.568	0.642	0.858
**WNT16**	1.005	0.981	1.016

Supplementary Fig.5 ***Gene expression of SNIP, PTCH1,*** **and** ***PPP1R10 in CD63-BCAR4 overexpressed cells.*** **A.**RT-PCR (left) and quantitative real-time RT-PCR (right) were used to compare mRNA expression of *SNIP*, *PTCH1*, and *PPP1R10* between control vector and CD3-BCAR4 overexpressed cells. **B**. Expression level of GLI2-target genes was examined by microarray.
