# Supplementary material for: A fusion of CD63–BCAR4 identified in lung adenocarcinoma promotes tumorigenicity and metastasis
Source: Br J Cancer. 2020 Nov 18;124(1):290–8. doi: 10.1038/s41416-020-01146-3 (PMC7782829; doi:10.1038/s41416-020-01146-3)
Supplement: Supplementary file 2 — Supplementary tables [file 41416_2020_1146_MOESM2_ESM.pdf]

**Table S1.** Information of NCCLUAD patients

| Sample ID | Histology | Tumor grade* | Gender | Age | Smoking | Pack-year | EGFR Mutation | KRAS Mutation | RNA-seq |
|---|---|---|---|---|---|---|---|---|---|
| NCCLUAD-002 | Lung adenocarcinoma | PD | M | 62 | Ever | 63 | Not detected | Not detected | |
| NCCLUAD-003 | Lung adenocarcinoma | PD | M | 59 | Ever | 45 | Not detected | Not detected | |
| NCCLUAD-004 | Lung adenocarcinoma | PD | F | 53 | Never | | Not detected | Not detected | |
| NCCLUAD-005 | Lung adenocarcinoma | MD | F | 60 | Never | | Not detected | Not detected | |
| NCCLUAD-006 | Lung adenocarcinoma | PD | F | 52 | Never | | Not detected | Not detected | |
| NCCLUAD-007 | Lung adenocarcinoma | PD | F | 44 | Never | | Not detected | Not detected | KIF5-RET |
| NCCLUAD-008 | Lung adenocarcinoma | - | M | 59 | Ever | 80 | Not detected | Not detected | |
| NCCLUAD-009 | Lung adenocarcinoma | PD | M | 54 | Ever | 6 | Not detected | Not detected | KIF5-RET |
| NCCLUAD-010 | Lung adenocarcinoma | MD | F | 53 | Never | | Not detected | Not detected | CD63-BCAR4 |
| NCCLUAD-011 | Lung adenocarcinoma | PD | M | 51 | Ever | 1 | Not detected | Not detected | |
| NCCLUAD-012 | Lung adenocarcinoma | PD | F | 77 | Never | | Not detected | Not detected | |
| NCCLUAD-014 | Lung adenocarcinoma | - | M | 40 | Ever | 4 | Not detected | Not detected | EML4-ALK |
| NCCLUAD-018 | Lung adenocarcinoma | PD | F | 63 | Never | | Not detected | Not detected | EML4-ALK |
| NCCLUAD-019 | Lung adenocarcinoma | MD | M | 34 | Never | | Not detected | Not detected | |
| NCCLUAD-020 | Lung adenocarcinoma | MD | M | 69 | Never | | Not detected | Not detected | EML4-ALK |
| NCCLUAD-023 | Lung adenocarcinoma | MD | F | 54 | Ever | 10 | Not detected | Not detected | |
| NCCLUAD-024 | Lung adenocarcinoma | PD | M | 43 | Never | | Not detected | Not detected | |
| NCCLUAD-030 | Lung adenocarcinoma | PD | M | 37 | Ever | 18 | Not detected | Not detected | |
| NCCLUAD-031 | Lung adenocarcinoma | PD | M | 51 | Ever | 6.7 | Not detected | Not detected | |
| NCCLUAD-032 | Lung adenocarcinoma | PD | M | 52 | Ever | 2 | Not detected | Not detected | |
| NCCLUAD-033 | Lung adenocarcinoma | PD | M | 49 | Ever | 10 | Not detected | Not detected | EML4-ALK |
| NCCLUAD-034 | Lung adenocarcinoma | PD | M | 53 | Ever | 0.25 | Not detected | Not detected | |
| NCCLUAD-038 | Lung adenocarcinoma | MD | F | 38 | Never | | Not detected | Not detected | ROS1-SLC43A2 |
| NCCLUAD-043 | Lung adenocarcinoma | MD | F | 53 | Never | | Not detected | Not detected | |
| NCCLUAD-045 | Lung adenocarcinoma | WD | F | 54 | Never | | Not detected | Not detected | KIF5B-REB |
| NCCLUAD-046 | Lung adenocarcinoma | MD | F | 55 | Never | | Not detected | Not detected | EML4-ALK |
| NCCLUAD-047 | Lung adenocarcinoma | MD | F | 55 | Never | | Not detected | Not detected | |
| NCCLUAD-049 | Lung adenocarcinoma | MD | F | 57 | Never | | Not detected | Not detected | |
| NCCLUAD-050 | Lung adenocarcinoma | - | F | 47 | Never | | Not detected | Not detected | |

* PD, poorly differentiated; MD, moderately differentiated; WD, well differentiated

**Table S2.** Differentially expressed genes between empty vector and CD63-BCAR4 identified by microarray

| Category | Gene Symbol | Fold change | | | Gene Name |
|---|---|---|---|---|---|
| | | CD63-BCAR4 Stable cell-1 | CD63-BCAR4 Stable cell-2 | BCAR4 Stable cell | |
| Cell migration | MMP1 | 8.722 | 7.810 | 0.661 | matrix metallopeptidase 1 |
| | LINC00473 | 7.024 | 3.935 | 2.282 | long intergenic non-protein coding RNA 473 |
| Cell differentiation | DCLK1 | 5.833 | 7.099 | 1.643 | doublecortin-like kinase 1 |
| Extracellular matrix | COL13A1 | 5.693 | 7.137 | 1.450 | collagen, type XIII, alpha 1 |
| Cell differentiation | DMBT1 | 5.634 | 7.108 | 1.606 | deleted in malignant brain tumors 1 |
| | ACOX2 | 5.002 | 5.973 | 0.901 | acyl-CoA oxidase 2, branched chain |
| Cell differentiation, Neurogenesis | CACNG7 | 4.616 | 6.732 | 0.937 | calcium channel, voltage-dependent, gamma subunit 7 |
| | ETV4 | 4.588 | 6.487 | 0.793 | ets variant 4 |
| | SH3TC1 | 4.516 | 3.610 | 0.806 | SH3 domain and tetratricopeptide repeats 1 |
| Extracellular matrix | TGM2 | 3.975 | 5.945 | 1.527 | transglutaminase 2 |
| | FAM196B | 3.968 | 2.767 | 1.370 | family with sequence similarity 196, member B |
| Inflammatory response | CHST1 | 3.832 | 6.199 | 0.746 | carbohydrate (keratan sulfate Gal-6) sulfotransferase 1 |
| Cell Proliferation | CyclinD1 | 3.822 | 4.067 | 1.028 | cyclin D1 |
| | SPRY4 | 3.705 | 4.839 | 1.156 | sprouty homolog 4 (Drosophila) |
| Apoptotic process, Cell differentiation, Neurogenesis | PCSK9 | 3.654 | 3.277 | 13.917 | proprotein convertase subtilisin/kexin type 9 |
| Cell differentiation, Neurogenesis | ETV5 | 3.514 | 5.426 | 1.669 | ets variant 5 |
| Angiogenesis | ANGPTL4 | 3.469 | 6.646 | 0.467 | angiopoietin-like 4 |
| | SCD | 3.421 | 4.286 | 5.813 | stearoyl-CoA desaturase (delta-9-desaturase) |
| | DMKN | 3.184 | 4.106 | 0.853 | dermokine |
| Cell Proliferation | RORA | 3.177 | 3.948 | 0.948 | RAR-related orphan receptor A |
| | GALNT14 | 3.168 | 3.329 | 0.639 | polypeptide N-acetylgalactosaminyltransferase 14 |
| | RNA5-8S5 | 3.132 | 2.085 | 0.816 | RNA, 5.8S ribosomal 5 |
| | MPP4 | 3.131 | 2.962 | 0.924 | membrane protein, palmitoylated 4 (MAGUK p55 subfamily member 4) |
| | MPP4 | 3.129 | 3.108 | 1.209 | membrane protein, palmitoylated 4 (MAGUK p55 subfamily member 4) |
| Immune response, Secretion | TLR4 | 3.129 | 3.461 | 1.213 | toll-like receptor 4 |
| | DMKN | 3.110 | 3.539 | 0.767 | dermokine |
| | MEG3 | 2.982 | 3.224 | 1.549 | maternally expressed 3 (non-protein coding) |
| | IL17RD | 2.979 | 4.362 | 2.145 | interleukin 17 receptor D |
| Secretion | CA9 | 2.954 | 8.423 | 0.813 | carbonic anhydrase IX |
| Cell migration | DOCK4 | 2.941 | 3.805 | 0.742 | dedicator of cytokinesis 4 |
| | KCNMA1 | 2.917 | 3.281 | 1.259 | potassium channel, calcium activated large conductance subfamily M alpha, member |
| | SEMA3A | 2.915 | 3.098 | 1.235 | sema domain, immunoglobulin domain (Ig), short basic domain, secreted, (semaphori |
| | GOLGA6C | 2.811 | 2.317 | 1.977 | golgin A6 family, member C |
| | SYNE3 | 2.795 | 2.814 | 1.484 | spectrin repeat containing, nuclear envelope family member 3 |
| | TMEM233 | 2.762 | 3.304 | 0.598 | transmembrane protein 233 |
| | SPRED3 | 2.762 | 3.756 | 1.153 | sprouty-related, EVH1 domain containing 3 |
| | WNK2 | 2.752 | 3.502 | 1.408 | WNK lysine deficient protein kinase 2 |
| | FLJ41170 | 2.735 | 2.922 | 1.808 | uncharacterized LOC440200 |

| | | | | | |
|---|---|---|---|---|---|
| Cell Proliferation | HMGA2 | 2.709 | 2.550 | 1.069 | high mobility group AT-hook 2 |
| | NIM1K | 2.700 | 6.135 | 1.219 | NIM1 serine/threonine protein kinase |
| | MLPH | 2.686 | 4.646 | 0.615 | melanophilin |
| | AGPAT9 | 2.674 | 3.216 | 1.117 | 1-acylglycerol-3-phosphate O-acyltransferase 9 |
| | NRG2 | 2.655 | 4.122 | 0.855 | neuregulin 2 |
| Cell differentiation, Neurogenesis | CDH4 | 2.641 | 2.864 | 0.982 | cadherin 4, type 1, R-cadherin (retinal) |
| | TUBBP5 | 2.639 | 4.991 | 1.024 | tubulin, beta pseudogene 5 |
| | STXBP5L | 2.598 | 2.679 | 2.051 | syntaxin binding protein 5-like |
| | LRP3 | 2.537 | 3.018 | 1.453 | low density lipoprotein receptor-related protein 3 |
| | IRX4 | 2.513 | 2.795 | 1.651 | iroquois homeobox 4 |
| | C2orf82 | 2.513 | 2.103 | 1.995 | chromosome 2 open reading frame 82 |
| Cell differentiation, Neurogenesis | DPYSL5 | 2.512 | 2.755 | 1.046 | dihydropyrimidinase-like 5 |
| | LYPD1 | 2.494 | 2.870 | 1.751 | LY6/PLAUR domain containing 1 |
| Cell Proliferation | STC1 | 2.485 | 4.824 | 1.029 | stanniocalcin 1 |
| | LINC00637 | 2.466 | 2.579 | 2.171 | long intergenic non-protein coding RNA 637 |
| Immune response | APLN | 2.463 | 4.369 | 1.117 | apelin |
| | LOC101929056 | 2.457 | 2.450 | 1.843 | uncharacterized LOC101929056 |
| | MEG8 | 2.447 | 3.255 | 1.438 | maternally expressed 8 (non-protein coding) |
| | CAMK2N1 | 2.428 | 3.450 | 1.080 | calcium/calmodulin-dependent protein kinase II inhibitor 1 |
| Cell migration | LAMA5 | 2.424 | 3.755 | 1.365 | laminin, alpha 5 |
| | CERS1 | 2.421 | 2.852 | 1.204 | ceramide synthase 1 |
| | FLJ32255 | 2.366 | 2.747 | 1.320 | uncharacterized LOC643977 |
| Cell cycle | SPRY1 | 2.316 | 5.727 | 0.653 | sprouty homolog 1, antagonist of FGF signaling (Drosophila) |
| | SLC1A1 | 2.289 | 3.896 | 1.103 | solute carrier family 1 (neuronal/epithelial high affinity glutamate transporter, system |
| Cell differentiation, Neurogenesis | DPYSL5 | 2.286 | 2.942 | 1.004 | dihydropyrimidinase-like 5 |
| | LOC284080 | 2.280 | 2.525 | 1.323 | uncharacterized LOC284080 |
| | DMKN | 2.276 | 2.712 | 0.868 | dermokine |
| Cell differentiation | DUSP6 | 2.266 | 2.786 | 1.089 | dual specificity phosphatase 6 |
| Cell Proliferation | BCL2 | 2.263 | 2.158 | 1.301 | B-cell CLL/lymphoma 2 |
| | NT5E | 2.262 | 2.801 | 1.421 | 5'-nucleotidase, ecto (CD73) |
| | STEAP1 | 2.256 | 3.632 | 0.735 | six transmembrane epithelial antigen of the prostate 1 |
| | LINC01433 | 2.252 | 2.944 | 1.489 | long intergenic non-protein coding RNA 1433 |
| | DPF3 | 2.248 | 2.404 | 1.043 | D4, zinc and double PHD fingers, family 3 |
| Cell cycle | RRAGD | 2.223 | 3.572 | 0.929 | Ras-related GTP binding D |
| | BTBD11 | 2.223 | 3.522 | 0.726 | BTB (POZ) domain containing 11 |
| Angiogenesis, Cell differentiation | GPR4 | 2.193 | 3.423 | 1.136 | G protein-coupled receptor 4 |
| | SLC12A7 | 2.182 | 2.346 | 1.181 | solute carrier family 12 (potassium/chloride transporter), member 7 |
| Cell differentiation, Neurogenesis | LDLR | 2.165 | 2.168 | 2.962 | low density lipoprotein receptor |
| Extracellular matrix | TGM2 | 2.159 | 2.107 | 1.121 | transglutaminase 2 |
| | DUSP4 | 2.151 | 2.198 | 1.019 | dual specificity phosphatase 4 |
| | CELF2 | 2.149 | 2.351 | 0.265 | CUGBP, Elav-like family member 2 |
| | PGF | 2.146 | 3.154 | 0.979 | placental growth factor |
| Immune response, Secretion | TLR4 | 2.145 | 2.357 | 0.823 | toll-like receptor 4 |
| Cell cycle, Cell death, Neurogenesis | BRSK2 | 2.129 | 2.655 | 1.006 | BR serine/threonine kinase 2 |

| | | | | | |
|---|---|---|---|---|---|
| | KANK3 | 2.126 | 2.190 | 1.012 | KN motif and ankyrin repeat domains 3 |
| Cell migration | EPHB3 | 2.123 | 2.429 | 0.645 | EPH receptor B3 |
| | NALCN | 2.109 | 2.372 | 1.328 | sodium leak channel, non selective |
| | ST3GAL6 | 2.096 | 2.975 | 1.301 | ST3 beta-galactoside alpha-2,3-sialyltransferase 6 |
| | ANO7 | 2.086 | 2.211 | 2.191 | anoctamin 7 |
| | THSD4 | 2.074 | 2.322 | 1.255 | thrombospondin, type I, domain containing 4 |
| | EPHA1-AS1 | 2.074 | 3.433 | 0.791 | EPHA1 antisense RNA 1 |
| Apoptotic process, Immune response | LY96 | 2.067 | 2.328 | 0.757 | lymphocyte antigen 96 |
| Cell differentiation, Neurogenesis | NDRG1 | 2.059 | 4.576 | 0.958 | N-myc downstream regulated 1 |
| Cell Proliferation | HMGA2 | 2.058 | 2.522 | 0.694 | high mobility group AT-hook 2 |
| | ARHGEF4 | 2.047 | 3.437 | 0.968 | Rho guanine nucleotide exchange factor (GEF) 4 |
| Cell Proliferation, Cell migration, Apoptotic process | DDIT4 | 2.044 | 4.488 | 1.357 | DNA-damage-inducible transcript 4 |
| | LINC01291 | 2.042 | 2.981 | 0.807 | long intergenic non-protein coding RNA 1291 |
| | SETD3 | 2.041 | 2.043 | 1.495 | SET domain containing 3 |
| | PRRT1 | 2.037 | 2.139 | 1.700 | proline-rich transmembrane protein 1 |
| | TMEM74B | 2.031 | 3.929 | 0.781 | transmembrane protein 74B |
| | ITGB8 | 2.023 | 2.619 | 1.560 | integrin, beta 8 |
| | SFRP1 | 2.020 | 3.473 | 1.631 | secreted frizzled-related protein 1 |
| Cell differentiation | SULF2 | 2.014 | 2.460 | 0.685 | sulfatase 2 |
| Cell cycle | RASSF2 | 2.012 | 3.251 | 0.915 | Ras association (RalGDS/AF-6) domain family member 2 |
| Cell cycle | INHBA | 0.500 | 0.493 | 0.821 | inhibin, beta A |
| | ARSG | 0.499 | 0.478 | 0.578 | arylsulfatase G |
| | LOC392364 | 0.493 | 0.320 | 0.373 | nuclear pore associated protein 1 pseudogene |
| | PRDM15 | 0.492 | 0.396 | 0.429 | PR domain containing 15 |
| | ARMCX4 | 0.490 | 0.315 | 0.234 | armadillo repeat containing, X-linked 4 |
| | SAMD11 | 0.484 | 0.487 | 1.013 | sterile alpha motif domain containing 11 |
| | LINC01124 | 0.471 | | | long intergenic non-protein coding RNA 1124 |
| Apoptotic process | TRIM39 | 0.471 | 0.464 | 0.473 | tripartite motif containing 39 |
| | SEPP1 | 0.470 | 0.304 | 0.226 | selenoprotein P, plasma, 1 |
| | DIO2 | 0.464 | 0.296 | 1.080 | deiodinase, iodothyronine, type II |
| | PLEKHA7 | 0.461 | 0.430 | 0.827 | pleckstrin homology domain containing, family A member 7 |
| Apoptotic process | PRUNE2 | 0.460 | 0.477 | 1.691 | prune homolog 2 (Drosophila) |
| | HCK | 0.459 | 0.237 | 0.554 | HCK proto-oncogene, Src family tyrosine kinase |
| | TBC1D22A | 0.459 | 0.288 | 0.385 | TBC1 domain family, member 22A |
| | KRT16P2 | 0.453 | 0.348 | 0.640 | keratin 16 pseudogene 2 |
| | C10orf120 | 0.447 | 0.497 | 0.362 | chromosome 10 open reading frame 120 |
| | ZCCHC6 | 0.443 | 0.317 | 0.526 | zinc finger, CCHC domain containing 6 |
| | GGTLC2 | 0.441 | 0.492 | 0.482 | gamma-glutamyltransferase light chain 2 |
| Cell migration | GREM1 | 0.441 | 0.434 | 0.687 | gremlin 1, DAN family BMP antagonist |
| Cell differentiation | SOX3 | 0.439 | 0.492 | 0.299 | SRY (sex determining region Y)-box 3 |
| | LOC284950 | 0.436 | 0.298 | 1.146 | uncharacterized LOC284950 |
| | STEAP4 | 0.427 | 0.162 | 0.202 | STEAP family member 4 |
| | PTGFR | 0.424 | 0.486 | 0.982 | prostaglandin F receptor (FP) |
| | ZNF236 | 0.419 | 0.471 | 0.566 | zinc finger protein 236 |

| | | | | | |
|---|---|---|---|---|---|
| | GGTLC1 | 0.418 | 0.465 | 0.417 | gamma-glutamyltransferase light chain 1 |
| | ITFG3 | 0.415 | 0.405 | 0.379 | integrin alpha FG-GAP repeat containing 3 |
| Extracellular matrix | RARRES2 | 0.409 | 0.444 | 1.087 | retinoic acid receptor responder (tazarotene induced) 2 |
| Cell Proliferation | PDZK1 | 0.401 | 0.437 | 0.942 | PDZ domain containing 1 |
| Cell Proliferation | PRDM1 | 0.399 | 0.463 | 1.151 | PR domain containing 1, with ZNF domain |
| Extracellular matrix | ADAMTS1 | 0.399 | 0.370 | 0.797 | ADAM metallopeptidase with thrombospondin type 1 motif, 1 |
| Extracellular matrix | EDIL3 | 0.391 | 0.400 | 0.846 | EGF-like repeats and discoidin I-like domains 3 |
| | LOC400743 | 0.389 | 0.498 | 0.266 | uncharacterized LOC400743 |
| Cell cycle | ZBTB49 | 0.389 | 0.292 | 0.372 | zinc finger and BTB domain containing 49 |
| | PTGFR | 0.384 | 0.481 | 0.936 | prostaglandin F receptor (FP) |
| | POU6F1 | 0.382 | 0.489 | 0.523 | POU class 6 homeobox 1 |
| Extracellular matrix | AEBP1 | 0.381 | 0.438 | 0.741 | AE binding protein 1 |
| Cell Proliferation | NR4A3 | 0.378 | 0.352 | 0.962 | nuclear receptor subfamily 4, group A, member 3 |
| Cell migration | GREM1 | 0.376 | 0.386 | 0.757 | gremlin 1, DAN family BMP antagonist |
| Cell migration | AMOT | 0.372 | 0.461 | 0.234 | angiomotin |
| Cell migration | CYP1B1 | 0.370 | 0.471 | 1.034 | cytochrome P450, family 1, subfamily B, polypeptide 1 |
| | lnc-STOM-2 | 0.367 | 0.328 | 0.255 | lnc-STOM-2:2 |
| | TMEM140 | 0.362 | 0.484 | 0.459 | transmembrane protein 140 |
| | NOSTRIN | 0.361 | 0.447 | 0.770 | nitric oxide synthase trafficking |
| Angiogenesis | ACKR3 | 0.360 | 0.473 | 0.782 | atypical chemokine receptor 3 |
| Cell Proliferation | ATOH8 | 0.359 | 0.400 | 1.052 | atonal homolog 8 (Drosophila) |
| Inflammatory response | NCR3 | 0.356 | 0.452 | 0.476 | natural cytotoxicity triggering receptor 3 |
| Cell cycle | FGF9 | 0.353 | 0.295 | 0.267 | fibroblast growth factor 9 |
| | PROC | 0.352 | 0.442 | 0.423 | protein C (inactivator of coagulation factors Va and VIIIa) |
| | lnc-CLIC6-1 | 0.350 | 0.420 | 0.398 | lnc-CLIC6-1:1 |
| Cell migration | CCL7 | 0.349 | 0.386 | 0.262 | chemokine (C-C motif) ligand 7 |
| | KRT42P | 0.346 | 0.329 | 0.585 | keratin 42 pseudogene |
| Aging | KRT14 | 0.343 | 0.358 | 0.604 | keratin 14, type I |
| | FAXC | 0.342 | 0.449 | 0.377 | failed axon connections homolog (Drosophila) |
| | MSL3 | 0.340 | 0.377 | 0.500 | male-specific lethal 3 homolog (Drosophila) |
| | LOC400553 | 0.339 | | 0.135 | uncharacterized LOC400553 |
| Cell differentiation, Cell death | KRT17 | 0.332 | 0.365 | 0.647 | keratin 17, type I |
| | C7orf69 | 0.331 | 0.268 | 0.842 | chromosome 7 open reading frame 69 |
| Cell differentiation | TRPC4 | 0.329 | 0.370 | 1.011 | transient receptor potential cation channel, subfamily C, member 4 |
| Secretion | CPLX2 | 0.327 | 0.458 | 0.389 | complexin 2 |
| | NIPAL4 | 0.327 | 0.466 | 0.246 | NIPA-like domain containing 4 |
| | CRYM | 0.326 | 0.247 | 0.368 | crystallin, mu |
| Cell migration | NRG2 | 0.325 | 0.496 | 0.427 | neuregulin 2 |
| | TPH2 | 0.322 | 0.214 | 0.172 | tryptophan hydroxylase 2 |
| | LOC100268168 | 0.319 | 0.384 | 0.391 | uncharacterized LOC100268168 |
| | LOC100129027 | 0.319 | 0.214 | 0.324 | uncharacterized LOC100129027 |
| Extracellular matrix | ADAMTS1 | 0.314 | 0.303 | 0.861 | ADAM metallopeptidase with thrombospondin type 1 motif, 1 |
| | FAR2 | 0.304 | 0.476 | 0.793 | fatty acyl CoA reductase 2 |
| Cell migration | PDE4D | 0.304 | 0.378 | 0.190 | phosphodiesterase 4D, cAMP-specific |

| | | | | | |
|---|---|---|---|---|---|
| | RDH16 | 0.295 | 0.269 | 0.274 | retinol dehydrogenase 16 (all-trans) |
| Cell differentiation | LIPN | 0.295 | 0.282 | 0.235 | lipase, family member N |
| | B3GALT4 | 0.294 | 0.411 | 1.203 | UDP-Gal:betaGlcNAc beta 1,3-galactosyltransferase, polypeptide 4 |
| Cell migration | S100A1 | 0.291 | 0.282 | 0.276 | S100 calcium binding protein A1 |
| | MUC5AC | 0.289 | 0.189 | 0.245 | mucin 5AC, oligomeric mucus/gel-forming |
| Immune response | C4BPB | 0.288 | 0.207 | 0.612 | complement component 4 binding protein, beta |
| | FAM230B | 0.283 | 0.331 | 0.173 | family with sequence similarity 230, member B (non-protein coding) |
| | AMPH | 0.278 | 0.374 | 0.846 | amphiphysin |
| Cell differentiation | EGR2 | 0.277 | 0.321 | 0.733 | early growth response 2 |
| | TMPRSS15 | 0.276 | 0.287 | 0.271 | transmembrane protease, serine 15 |
| | TRABD2B | 0.274 | 0.454 | 0.576 | TraB domain containing 2B |
| Cell differentiation, Neurogenesis | GPR37L1 | 0.265 | 0.469 | 0.781 | G protein-coupled receptor 37 like 1 |
| | ECEL1P2 | 0.263 | 0.264 | 0.746 | endothelin converting enzyme-like 1, pseudogene 2 |
| Cell migration | NRP2 | 0.260 | 0.323 | 0.446 | neuropilin 2 |
| | MSC | 0.258 | 0.414 | 0.815 | musculin |
| | SPG20-AS1 | 0.253 | 0.315 | 0.464 | SPG20 antisense RNA 1 |
| Cell Proliferation | IGFN1 | 0.246 | 0.236 | 0.705 | immunoglobulin-like and fibronectin type III domain containing 1 |
| | CAMK1G | 0.243 | | 0.225 | calcium/calmodulin-dependent protein kinase IG |
| Cell cycle | PAX6 | 0.236 | 0.381 | 1.077 | paired box 6 |
| Cell migration | OLR1 | 0.233 | 0.227 | 1.032 | oxidized low density lipoprotein (lectin-like) receptor 1 |
| Extracellular matrix | ABI3BP | 0.231 | 0.219 | 0.864 | ABI family, member 3 (NESH) binding protein |
| | OR10J5 | 0.226 | 0.165 | 0.150 | olfactory receptor, family 10, subfamily J, member 5 |
| | lnc-ENPP1-2 | 0.223 | 0.276 | 0.208 | lnc-ENPP1-2:1 |
| | CMA1 | 0.220 | | | chymase 1, mast cell |
| Cell migration | NRP2 | 0.217 | 0.340 | 0.398 | neuropilin 2 |
| | EPDR1 | 0.216 | 0.325 | 0.358 | ependymin related 1 |
| | CNN1 | 0.213 | 0.251 | 0.267 | calponin 1, basic, smooth muscle |
| Cell migration | SAA2 | 0.201 | 0.405 | 1.237 | serum amyloid A2 |
| Cell migration | SAA4 | 0.200 | 0.426 | 1.362 | serum amyloid A4, constitutive |
| | CPE | 0.198 | 0.247 | 0.182 | carboxypeptidase E |
| Cell migration | EDN2 | 0.195 | 0.289 | 0.512 | endothelin 2 |
| Immune response | ENPP2 | 0.195 | 0.334 | 0.370 | ectonucleotide pyrophosphatase/phosphodiesterase 2 |
| Extracellular matrix | ABI3BP | 0.193 | 0.219 | 0.939 | ABI family, member 3 (NESH) binding protein |
| Cell cycle | PAX6 | 0.188 | 0.379 | 1.321 | paired box 6 |
| | PCDHB5 | 0.184 | 0.384 | 0.197 | protocadherin beta 5 |
| Cell Proliferation | DUSP2 | 0.179 | 0.156 | 0.655 | dual specificity phosphatase 2 |
| Neurogenesis | NPTX1 | 0.178 | 0.203 | | neuronal pentraxin I |
| Extracellular matrix | COL5A2 | 0.158 | 0.204 | 0.171 | collagen, type V, alpha 2 |
| | MAP3K14-AS1 | 0.154 | 0.208 | 0.189 | MAP3K14 antisense RNA 1 |
| | CACNA1G-AS1 | 0.153 | 0.259 | 1.211 | CACNA1G antisense RNA 1 |
| | MAN1A1 | | 0.140 | 0.452 | mannosidase, alpha, class 1A, member 1 |
| | KANK4 | | | 0.941 | KN motif and ankyrin repeat domains 4 |
